# The internationalization of human microbiome research

**DOI:** 10.1016/j.mib.2019.09.012

**Published:** 2019-08

**Authors:** Ana Maria Porras, Ilana Lauren Brito

**Affiliations:** Meinig School of Biomedical Engineering, Cornell University, Ithaca, NY, 14850, United States

## Abstract

The human microbiome has now been linked with myriad diseases, yet most of this research has been conducted on American and European populations that make up only 1/6th of the world’s population. With growing recognition that human microbiomes differ tremendously across global populations, it is especially important to understand how these compositional differences impact health outcomes. Recent advances in infectious disease and malnutrition research have demonstrated the potential for microbiome-based strategies to address the biggest challenges in global health. This review highlights major advances toward understanding microbiome diversity across the world and its contributions to disease, and outlines key questions, challenges, and opportunities to broaden the scope of and promote inclusivity within microbiome research.

**Current Opinion in Microbiology** 2019, **50**:50–55

This review comes from a themed issue on **Microbiota**

Edited by **Karen Guillemin** and **Julia A Segre**

For a complete overview see the Issue and the Editorial

Available online 2nd November 2019

**https://doi.org/10.1016/j.mib.2019.09.012**

1369-5274/© 2019 The Authors. Published by Elsevier Ltd. This is an open access article under the CC BY license (http://creativecommons.org/licenses/by/4.0/).

In the past decade, the human microbiome has been associated with alterations in insulin sensitivity, cardiovascular disease, behavioral disorders, susceptibility to allergies and infection, and even cancer treatment outcomes in populations in North America and Europe [1]. Yet, while we have learned that human gut and oral microbiomes are categorically different across the globe [2, 3, 4, 5, 6], research that links microbiota to health concerns of populations in the rest of the world has been noticeably lagging. The development of microbial therapeutics is imminent and may have relevance for major global health problems such as malnutrition, resilience to infection, and the growing threat of obesity and other metabolic disorders. To achieve this goal, it is first imperative to better understand whether reported microbiome effects are truly ‘global’.

## Major efforts to characterize microbiomes around the world

The first large-scale studies of the human microbiome, performed on metropolitan Americans and Europeans, exposed tremendous diversity between individuals’ microbiomes [7,8]. It is now clear that the initial interpersonal differences identified within these populations are small relative to global variation in the human microbiome [2, 3, 4, 5,9,10]. Compared to their American and European counterparts, the microbiomes of populations in low-income and middle-income countries (LMICs) are consistently more diverse and enriched in specific phylogenetic groups [6]. Motivated by these observations, a growing number of countries, including India [11] and China [12], have launched their own nation-wide microbiome initiatives. These endeavors will continue to expand our recognition of geographic variability in the microbiome.

**Figure 1:**
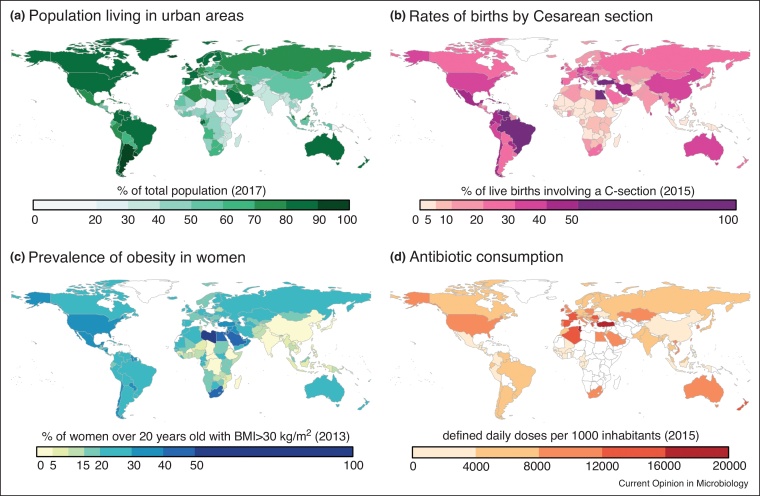
Global indicators of factors found to associate with microbiome composition in at least one population. **(a)** Urbanization data obtained from the United Nations Population Division [21]. **(b)** Cesarean section usage compiled by Boerma *et al.* [22]. **(c)** Prevalence of obesity in women over 20 years old reported by the Global Burden of Disease Consortium [23]. **(d)** Antibiotic consumption reported by the Center for Disease Dynamics, Economics & Policy [24]. All data were obtained directly from the sources and plotted using the ‘rworldmap’ package in R. Data were not available for the countries colored in white.

There are many factors at play that could drive or explain the differences observed across populations including host genetics [13], diet [4], birth and breastfeeding practices [14, 15, 16], socio-economic status [17], urbanization [18], agricultural dependence [19,20], and antibiotic usage [2,17]. Now that these initial differences have been characterized, the field can, and should, study these issues along wider spectrums that are representative of a wider proportion of the world’s population. Many of these factors, such as urbanization [21], the use of cesarean sections [22], antibiotic consumption [23], and prevalence of obesity [24] (Figure 1), do not follow simple trends that can be easily divided or explained away across the high-income versus middle-income and low-income divide. To address these nuances, recent studies have explicitly sought to explore the human gut microbiomes along a range of urban settings in Latin America [17] and Africa [18] that more closely approximate the living conditions of many populations in LMICs (Figure 1a). This type of approach will be necessary to characterize the microbiome across the full range of the human experience.

The complexity of the microbiomes within even one population make it difficult to understand its varied impacts on host health. The current paradigm under which the effects of individual or small groups of bacteria are studied in isolation has delivered conflicting reports. For example, LMIC microbiomes are rich in *Prevotella* species, which are hypothesized to be the result of a plant-based fiber-rich diet and beneficial to the host [4,6,9,25]. However, these bacteria have also been associated with a wide range of positive and negative health outcomes [26,27]. Similarly, many *Proteobacteria*, highly prevalent in LMIC microbiomes, are primarily known as opportunistic pathogens and have been proposed as markers of dysbiosis [28]. Furthermore, mucus-degrading species that are often found in high-income countries (HICs) and rarely in LMICs [19,25], such as those of the *Bacteroides* genus and *Akkermansia muciniphila,* have been linked to inflammation [6]. At the same time, *A. muciniphila* has been reported to improve metabolism and protect against obesity and diabetes in mice [29]. These results motivate the growing call [5,30] to investigate bacterial functional roles from an ecological perspective within the specific context of each population’s microbiomes.

Much remains to be gained from diversifying human microbiome research by increasing the representation of LMICs within studies. A series of recent culture-independent metagenomic approaches reconstructed thousands of previously unidentified bacterial genomes in populations outside of the United States and Europe [31, 32, 33]. Collectively, these studies greatly expanded the repertoire of known species of the human microbiome, particularly for underrepresented populations in this field. When comparing reference genomes to the new metagenome-assembled genomes (MAGs), one study reported an increase in metagenomic read mappability from 75% to 95% in the gut microbiomes of Westernized populations compared to an increase from 45% to 85% in those of non-Westernized populations [31]. In subsets of these populations read assignment improved even more dramatically; specifically, classification of African and Latin American samples improved by 200% [32].

The generated metagenome assemblies also revealed the presence of eukaryotic organisms including parasites, fungi, and protozoans. While ubiquitous across all human microbiomes, these organisms are particularly abundant in LMICs due to differences in diet, access to clean water, hygiene habits, and antibiotic use, among other causes [34]. A few seminal studies have reported the association of fungal gut alterations with Crohn’s disease and colitis [35,36] in mice, and asthma in both American and Ecuadorian cohorts [37,38]. These results highlight the need to continue to study the interactions between eukaryotes and bacteria in the gut, as well as their influence over gut health and the immune system. Human microbiome research is far from a saturation point; arriving at a truly universal understanding of the human microbiome and its effect on host health will require global initiatives that include populations, socioeconomic and environmental factors, and/or diseases beyond those relevant exclusively to high-income countries. Several key outstanding questions in global microbiome research are summarized in Box 1.

Box 1 **Outstanding questions in global microbiome research**

What features of the microbiome are universal and which are more population-specific? Importantly, what is their effect on human health?

What is the role of eukaryotic organisms in the gut microbiome? Why is the prevalence of these organisms higher in certain populations and what is their effect on the immune system?

Are local microbiomes more or less resilient to local infections? Or are there universal features that make microbiomes more resilient regardless of composition? How does a wider exposure to pathogens early in life affect microbiome resiliency?

Are the influences of the microbiome on non-communicable diseases such as obesity and atherosclerosis the same in LMICs as observed in studies in high-income countries?

What are the geographic/population-specific contributions to inflammatory bowel disease (IBD), which is growing in prevalence around the world?

How does access to antibiotics (e.g. prescription, over-the-counter, agricultural, secondary exposure through the water supply) alter microbiome function?

Where should fecal slurries be sourced from for fecal microbiota transplantation trials?

How does growing access to processed foods alter population-specific microbiome composition and function?

What is the effect of human migration on the microbiome, specifically related to alterations in diet, and its impact on immune signaling?

How will a broader study population improve our understanding of genetic versus environmental contributions to microbiome composition?

Alt-text: Box 1

## Diseased-focused efforts to examine microbial contributors to host health

A valuable and natural approach to increase the participation of LMIC researchers in microbiome science would be to broaden its scope to include a wide variety of diseases that affect local populations, Research over the last decade has demonstrated that the microbiome plays an important role in building resistance to infectious diseases including lower respiratory tract infections [39], malaria [40], HIV [41], tuberculosis [42], and diarrheal diseases [43]. Given that these particular diseases lie within the top 10 leading causes of death in low income countries [44], microbiome-based therapeutics have the potential to significantly impact global health. For example, we now know that high abundance of *Gardnerella vaginalis* in the vaginal microbiome is linked to low efficacy of Tenofovir, a microbicide commonly used to prevent HIV infection [45]. In another case, an oral synbiotic preparation (with *Lactobacillus plantarum* as the probiotic and fructooligosaccharide as the prebiotic) was sufficient to significantly reduce sepsis and death in rural Indian newborns [46].

A critical factor to consider in many global settings is that patients are often exposed to more than one infectious agent at a time and treatment for one pathogen can have consequences for other infections. Antiretroviral therapy shifts the gut microbiome of HIV-infected patients toward the production of short chain fatty acids, which then increase susceptibility to tuberculosis infection [47]. The infection itself can also change the composition of the microbiome; these changes, in turn, alter the subsequent immune response to a secondary infection, as is often the case in respiratory infections [48]. Helminth and other intestinal parasites are endemic in LMICs and known to affect the gut microbiome [49]; however, the impact of the helminth-modified microbiome on vulnerability to other infections has not been explored. Similarly, little is known about the interactions between the human microbiome and neglected tropical diseases such as Chagas disease, rabies, and leishmaniasis, which collectively affect a sixth of the world’s population and are often caused by protozoans and other eukaryotic organisms [50]. Thus, more studies investigating the mechanisms behind these relationships and involving human cohorts that are particularly vulnerable to these infections will be necessary to unravel the complex interactions between the microbiome and infectious diseases in a global context.

Over the last few decades the prevalence of non-communicable diseases (NCDs) in LMICs has increased at an accelerated pace, particularly in middle-income countries, where these disorders have overtaken infectious diseases as the leading causes of the death [44]. The gut microbiome has been associated with most of these NCDs, including chronic obstructive pulmonary disease, diabetes, Alzheimer’s disease, obesity, and cardiovascular disease [51]. Yet, the association between the microbiome and these NCDs in LMIC populations has been scarcely studied and directly extrapolating from existing data will not be straight forward given the conflicting disease associations of bacteria present in differential abundance between populations. For example, *Prevotella* species associated with inflammatory bowel disease (IBD) in American populations also happen to be highly abundant in LMICs with low prevalence of this disease [6,27]. Similarly, in some parts of the world, like the Pacific Islands, plant-based diets [52], commonly associated with a healthy microbiome and metabolic state, co-occur with high rates of obesity and diabetes [44]. These apparent contradictions will not be resolved without a global approach that includes these underrepresented populations.

The identification of microbial contributors to host health at a global scale will depend on the ability to reach the populations most affected by these diseases and a holistic interpretation of the results. Such an approach applied in Malawi and Bangladesh has allowed the identification of microbiota immaturity as a major contributor to severe acute malnutrition and the design of treatments other than traditional nutritional intervention [53] such as the use of microbiota-directed complementary food [54,55]. Globalization adds another layer of complexity to the relationship between the microbiome and host health. The field must continue to explore the influence of migration [9] and temporary travel [56,57] on the composition of the microbiome and the resulting effects on susceptibility to both infectious and non-communicable diseases. As the number of populations with characterized healthy and disease-associated microbiomes increases, it will become easier to identify patterns and shifts in composition or functional profiles that are common across the world or unique to specific populations. Only then will it be possible to design microbiome-based health interventions applicable to most of the world’s inhabitants.

## Opportunities and challenges for broadening the coalition of microbiome researchers

International research will be strengthened by research practices that leverage local expertise and broadly available resources. Microbiome research faces significant capacity building challenges for widespread adoption, such as the need for cold chain transport of samples, relatively expensive extraction methods, sequencing and access to computing resources. Developing multi-national cohort studies also necessitates common criteria, which may be difficult to obtain across populations with varying diagnostic capabilities and disease emphasis. Furthermore, sequencing technologies have fully eclipsed cheaper techniques such as pulsed-field gel electrophoresis or restriction fragment length polymorphism analysis. Yet, some of these barriers will be reduced over time because of centralized sequencing facilities and computational clusters, and overall reductions in the cost of sequencing. Similarly, open access to scientific journals, the ubiquity of open-source computing packages (i.e. mothur, QIIME), and the availability of online training and tutorials will broaden access to and promote global participation in microbiome research.

Cross-cultural studies and inclusive research practices are valuable to test and challenge our assumptions of the role of the microbiome in human health. These studies are imperative to avoid generalizations such as the dismissal of non-Western medicine, hype around specific diets or supplements, or the romanticization of ‘natural’, ancestral microbiomes. These tendencies have surfaced within the public understanding of probiotics and the gut microbiome, and with the broad adoption of the lost Eastern medicine practice of Fecal Microbiota Transplantation (FMT) [58]. Comparative international gut microbiome research will help elucidate underlying universal mechanisms of disease or population-specific taxa that interact with host genetics, health history, and cultural practices to modulate disease outcomes.

Shifts in our perceptions of ‘healthy’ and ‘dysbiotic’ microbiomes, and ‘probiotic’ and ‘pathogenic’ bacteria are also reflected in the nomenclature shift from the Hygiene Hypothesis to the Old Friends Hypothesis [59]. The former stated that early exposure to ‘germs’ was crucial for immune system maturation; the latter, in contrast, argues that the important exposures during development are not to infectious agents but rather to the microbes present during ancestral times that have co-evolved with the human immune system. As a result of this shift, efforts have emerged to correct exposures that reflect Western practices associated with negative health outcomes, such as the ‘bacterial baptism’, that is, swabbing newborns with vaginal microbiome contents to counteract the lack of exposure to vaginal microbes in caesarean sections [60]. Nonetheless, these new practices must be evaluated for safety and efficacy.

Another important shift in global microbiome research is the integration of One Health approaches, in which microbial transfers between humans, animals, plant,s and the environment are evaluated holistically [61]. Traditionally, these transfers have been considered primarily in the identification of pathogens and pathogenic sources. However, recent studies have demonstrated that close contact with livestock, for example, leads to sharing of some commensal microbes between farmers and pigs, and children and cows in Denmark [62] and Kenya [63], respectively. These interactions can lead not only to the transfer of whole microbes, but also to the transfer of mobile genes. Gene sharing of antibiotic resistance genes across human, animal, and soil communities in two Latin American settings has already been reported [17].

Microbiome research is poised to have a large influence on human health. Moreover, low opportunity costs may be associated with the development of low-risk probiotics, prebiotics, and FMT treatments to combat infections, malnourishment, and metabolic disorders, and even improve vaccine efficacy. In recent years, microbiome research has been embraced by the Gates Foundation and the Wellcome Trust, among other international research agencies, with the goal of promoting inclusionary international microbiome research and broadening the constituency that reap these benefits. Building international and cross-cultural research teams to work in this space will fast-track identifying microbiome-based treatments that are either specific to each population or universally efficacious.
